# *BcMtg2* is required for multiple stress tolerance, vegetative development and virulence in *Botrytis cinerea*

**DOI:** 10.1038/srep28673

**Published:** 2016-06-27

**Authors:** Wenyong Shao, Yu Zhang, Jin Wang, Chiyuan Lv, Changjun Chen

**Affiliations:** 1 College of Plant Protection, Nanjing Agricultural University, Nanjing, 210095, China

## Abstract

In *Saccharomyces cerevisiae*, the *Mtg2* gene encodes the Obg protein, which has an important function in assembling ribosomal subunits. However, little is known about the role of the Obg GTPase in filamentous fungi. In this study, we identified an *Mtg2* ortholog, *BcMtg2*, in *B. cinerea*. The *BcMtg2* deletion mutant showed a defect in spore production, conidial germination and sclerotial formation. Additionally, the mutant increased sensitivity to various environmental stresses. The *BcMtg2* mutant exhibited dramatically decreased virulence on host plant tissues. *BcMtg2* mutant showed increased sensitivity to osmotic and oxidative stresses, and to Congo red (cell wall stress agent). In the yeast complement assay, growth defects of yeast BY4741ΔMTG2 mutant were partly restored by genetic complementation of *BcMtg2* under these environmental stresses. Additionally, compared with the parental strain and complement strain, the *BcMtg2* deletion mutant displayed a minor glycerol response to osmosis stress. These defective phenotypes were recovered in the complement strain *ΔBcMtg2C*, which was created by adding the wild-type *BcMtg2* gene to the *ΔBcMtg2* mutant. The results of this study indicate that *BcMtg2* has a necessary role in asexual development, environmental stress response and pathogenicity in *B. cinerea*.

GTPases have significant roles in many cell processes, including cell signaling, protein modification, cell reproduction, membrane permeability and cell growth. Furthermore, GTPases are conserved in many types of organisms, including eukaryotes and prokaryotes^1^,^2^,^3^,^4^. All organisms possess many conserved GTPases that are predicted to play roles in transcription^5^. Several of these GTPases have direct evidence for a role in ribosome function^6^,^7^,^8^. Prokaryotic GTPases include Era and Obg. The Era GTPase deletion mutant showed a decreased size of the ppGpp pool, had disturbed carbon metabolism and presented a polykaryon phenotype^9^,^10^,^11^. The Obg GTPase is another member of the prokaryotic GTPase superfamily. The Obg subfamily of GTPases has been sequenced and identified in all organisms to date^12^. Obg GTPase proteins have a conserved GTP-binding domain, indicating that they generate a general pattern of modification. Bacterial and mitochondrial Obg GTPase proteins may be orthologous because they are conserved throughout their protein lengths compared with other eukaryotic Obg GTPase proteins that have significantly distinct amino acid sequences except for the GTP-binding domain^12^. Previous studies have shown that various organisms have an Obg GTPase G-2 modification, including bacteria and humans^1^. In *Bacillus subtilis*, Obg GTPase proteins play an important role in original spore formation and vegetative growth^13^,^14^. In addition, previous research has shown that Obg GTPase proteins have an important function in regulating the σ^B^ transcription factor in *B. subtilis*^15^. Furthermore, in *Escherichia coli*, ObgE is necessary for chromosome disjunction^1^. In *Caulobacter crescentus*, the Obg family gene *CgtA* is essential for growth^16^. In *Arabidopsis thaliana*, the AtObgC, a homolog of the bacterial Obg GTPase gene, has an indispensible function in the synthesis of chloroplast proteins in early embryo development^17^. In *Saccharomyces cerevisiae*, each characteristic Obg GTPase protein seems to have a particular effect on ribosome function. The fourth *S. cerevisiae* mitochondrial Obg GTPase protein is encoded by YHR168W and is hereafter named *Mtg2* for mitochondrial GTPase 2^12^. *Mtg2* is indispensable for assembling the yeast mitochondrial ribosome^18^. *Mtg2* deletion mutations have defects in mitochondrial translation, which indicates that *Mtg2* plays an important role in mitochondrial ribosome function^12^. The protein encoded by the gene is a member of the Obg GTPase family, which plays a role in ribosomal assembly via binding the majority ribosomal subunit^19^. Thus, these studies have indicated that various Obg GTP proteins perform different functions in specific organisms. In our study, we analyzed the roles of the *Mtg2* homologous gene *BcMtg2* in *Botrytis cinerea*, this knowledge provides information for designing novel fungicides to control gray mold caused by *B. cinerea* and exploring the function of *BcMtg2* in *B. cinerea*.

*B. cinerea* causes gray mold which is a widespread and serious plant disease throughout the world^20^. Most plant organs, including leaves, shoots, bulb, flowers, and root tubers, are sensitive to *B. cinerea* infection. In varied environmental conditions, *B. cinerea* uses various patterns to infect its multiple hosts. In addition, *B. cinerea* can survive as conidia, sclerotia and mycelia for a period of time^21^. Therefore, controlling gray mold presents many challenges. Understanding the biology and host-pathogen interactions of *B. cinerea* is valuable for the discovery of an efficient strategy to control gray mold. A whole-genome search uncovered that *B. cinerea* an orthologue of *Mtg2* (hereafter named *BcMgt2*). Base on the previous research, we hypothesized that *BcMtg2* might own important effect on vegetative differentiation and virulence in *B. cinerea*. To clarify this hypothesis, the major objective of this study was to analyse the genetic, biological and biochemical function of *BcMtg2* using target gene deletion strategy. Our study revealed that *BcMtg2* plays significant role in regulating the asexual reproduction, vegetative growth, various stresses response and virulence in *B. cinerea.*

## Results

### Identification of the *BcMtg2* gene

Using the amino acid sequence of the *S. cerevisiae Mtg2* protein, *BcMtg2* was identified in *B. cinerea* using a BLASTP search in the *Botrytis cinerea* genome database (http://www.broad.mit.edu). The nucleotide sequence of *BcMtg2* is 1653 bp in size. The protein encoded by *BcMtg2* contains 550 amino acids. The predicted amino acid sequence indicated no introns in *BcMtg2*. An analysis of domains in the *BcMtg2* protein by SMART showed that *BcMtg2* includes two conserved domains: a Mg^2+^/GTP binding site in the N-terminus and a switch region in the C-terminus (Fig. S1).

### *BcMtg2* regulates vegetative growth and asexual reproduction in *B. cinerea*

**Figure 1:**
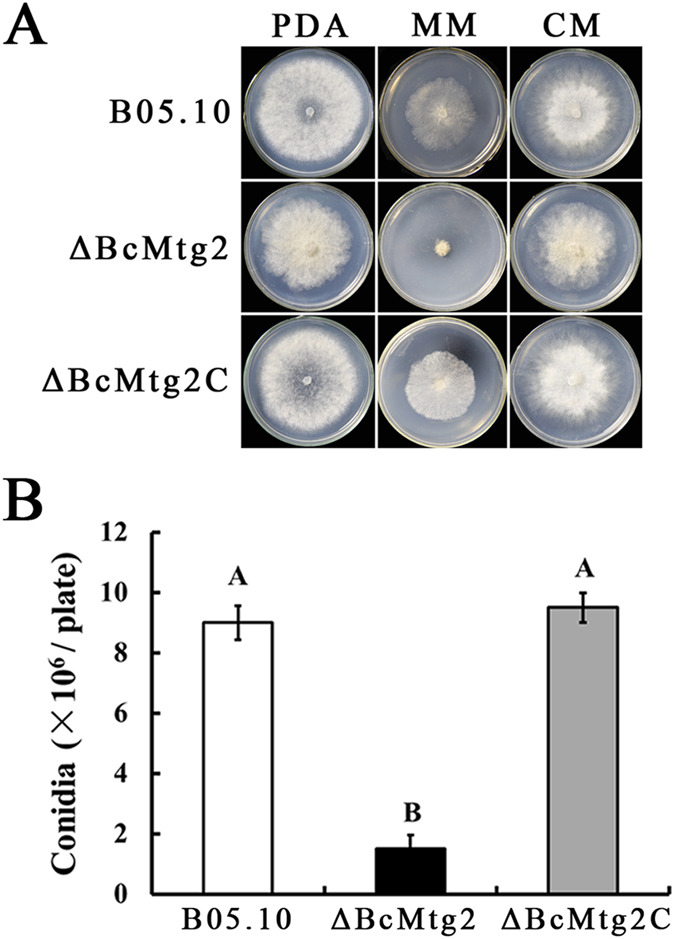
Function of *BcMtg2* on *Botrytis cinerea* growth and conidiation. (**A**) B05.10, *ΔBcMtg2* and *ΔBcMtg2C* were grown on PDA, MM or CM at 25 °C for 3 days. (**B**) The number of conidia produced by each strain on PDA plates. Bars denote standard errors from three experiments. Values on the bars followed by the same letter are not significantly different at P = 0.05.

**Figure 2:**
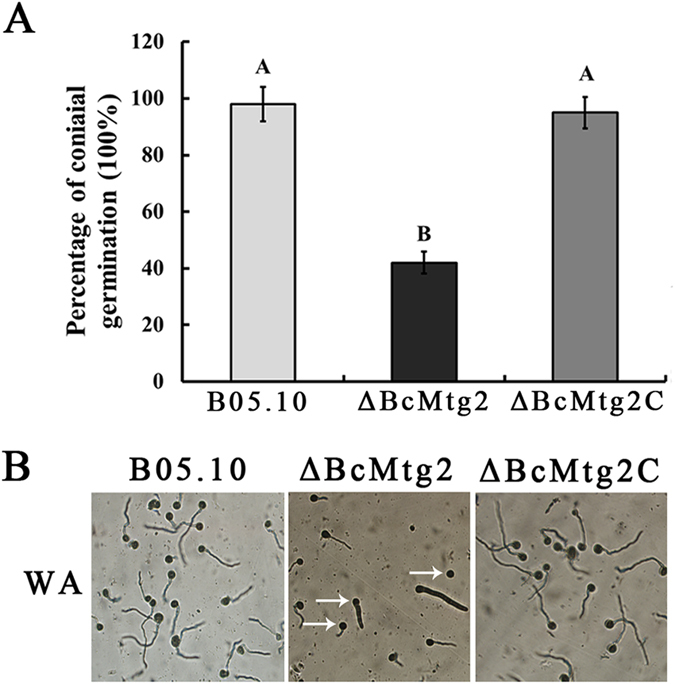
Effects of *BcMtg2* deletion on conidial germination (**A**) Conidial germination rate of each strain after cultivated on Water-Agar media at 25 °C for 15 h. Bars denote standard errors from three experiments. Values on the bars followed by the same letter are not significantly different at P = 0.05. (**B**) Germinal tube morphology of each strain after incubated on Water-Agar media at 25 °C for 15 h. Conidia with short and abnormal germ tubes are indicated by arrows.

On either complete medium (CM) or potato dextrose agar medium (PDA), the mycelial growth rate of the *BcMtg2* deletion mutant (*ΔBcMtg2*) was similar to the parental strain B05.10 and the complement strain *ΔBcMtg2*C. However, on minimal medium (MM), *ΔBcMtg2* grew more slowly than the wild-type strain B05.10 and the complement strain *ΔBcMtg2*C (Fig. 1A). In conidiation assay, *ΔBcMtg2* produced significantly fewer conidia than B05.10 and *ΔBcMtg2C* on PDA plate cultivated for 10 days (Fig. 1B). These results showed that *BcMtg2* was involved in conidiation in *B. cinerea.* The germination rate of conidia in *ΔBcMtg2* was reduced by 56%, compared with B05.10 and *ΔBcMtg2C* incubated for 15 h on Water-Agar medium (Fig. 2A). In addition, the germ tubes of the *BcMtg2* deletion mutant were malformed (Fig. 2B). These results showed that *BcMtg2* had an important role in mycelia growth and asexual reproduction in *B. cinerea*.

### Sensitivity of *B. cinerea* response to various stresses is regulated by *BcMtg2*

**Figure 3:**
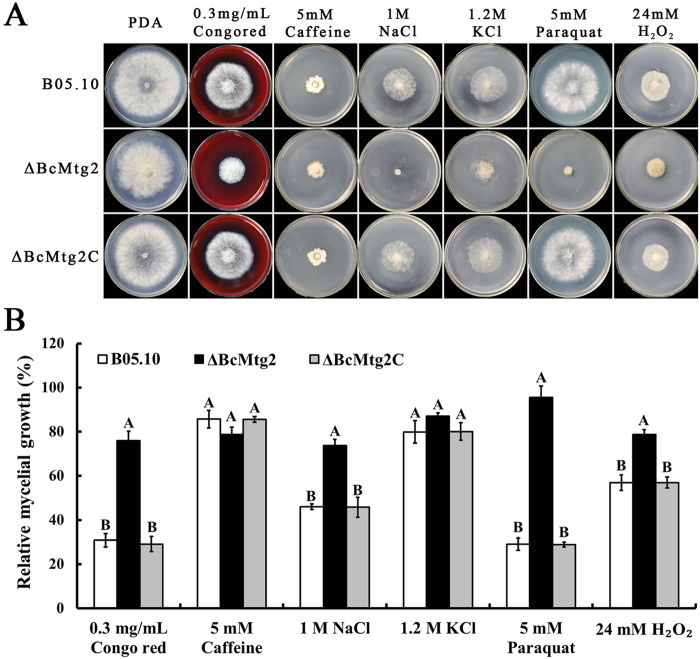
Sensitivity of B05.10, *ΔBcMtg2* and *ΔBcMtg2C* to multiple stress. (**A**) Comparisons were performed on PDA medium modified with Congo red, caffeine, NaCl, KCl, paraquat and H_2_O_2_ at the content indicated in the figure. (**B**) Inhibition of mycelial growth was analyzed after each strain was incubated for 3 days on PDA supplement with different compound as described in the figure. Bars denote standard errors from three experiments. Values on the bars followed by the same letter are not significantly different at P = 0.05.

**Figure 4:**
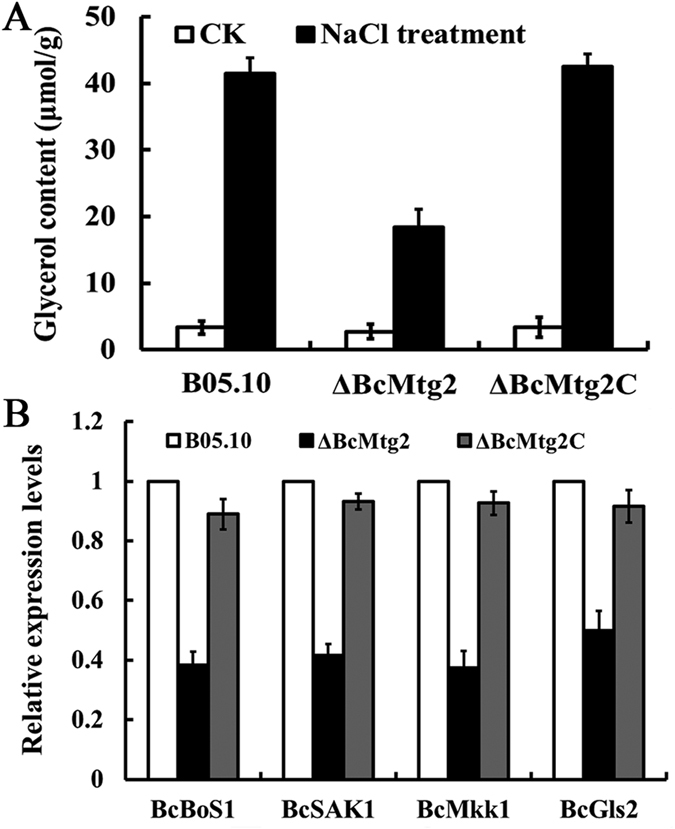
(**A**) Glycerol content in mycelia of B05.10, *ΔBcMtg2* and *ΔBcMtg2C*. Bars denote standard errors from three repeated experiments. (**B**) Expression levels of osmotic stress relation genes (*BcBoS1*, *BcSAK1*) and cell wall relation genes (*BcMkk1* and *BcGls2*) in each strain. RNA samples were isolated from mycelia treated with NaCl (1.0 M) and Congo red (0.3 mg ml^−1^) for 2 h respectively. Bars denote standard errors from three repeated experiments.

To explore the role of *BcMtg2* in *B. cinerea* response to multiple stresses, we measured the sensitivity of the wild-type strain B05.10, the *BcMtg2* deletion mutant, and the complemented strain *ΔBcMtg2C* to cell wall-damaging agents, osmotic stresses and oxidative pressure. Compared with the wild-type strain and the complemented strain, the sensitivity of *ΔBcMtg2* to osmotic stress was significantly increased (Fig. 3A,B). Previous studies have indicated that glycerol content plays a significant role in the regulation of fungus response to osmotic stress^22^,^23^. Thus, we measured glycerol accumulation in the parental strain B05.10, the *BcMtg2* deletion mutant and the complement strain *ΔBcMtg2C*. The result indicated that the parental strain B05.10, *ΔBcMtg2* and *ΔBcMtg2C* generated little glycerol without osmotic stress. However, the glycerol content of the parental strain B05.10 and the complement strain *ΔBcMtg2C* were significantly increased compared with that of the *BcMtg2* deletion mutant under osmotic stress conditions (Fig. 4A). Moreover, the previous study shown that the histidine kinase, *BcBoS1* and the mitogen-activated protein kinase, *BcSAK1* affect the sensitivity of *B. cinerea* responses to osmotic pressure^24^. The real-time PCR analysis showed that the transcription levels of the two genes were significantly decreased in *ΔBcMtg2* compared with that in B05.10 and *ΔBcMtg2C* under osmotic stress conditions (Fig. 4B). In addition, compared with B05.10 and *ΔBcMtg2*C, *ΔBcMtg2* presented increased sensitivity to the cell-damaging agent (Congo red) and to oxidative pressure (Fig. 3A,B). Previous studies had shown that *Mkk1* and *Gls2* were core component genes of cell wall completion in *S. cerevisiae*^25^. To understand the function of *BcMtg2* in cell wall completion in *B. cinerea*, the transcription levels of *BcMkk1* and *BcGls2* in each strain were analyzed. Compared with the parental strain B05.10 and the complement strain *ΔBcMtg2C*, the expression levels of the two genes were significantly decreased in *ΔBcMtg2* in response to Congo red (Fig. 4B), indicating that *BcMtg2* involved in various environmental stress response in *B. cinerea*.

### *BcMtg2* is involved in the sensitivity of *B. cinerea* response to cell wall lyase

**Figure 5:**
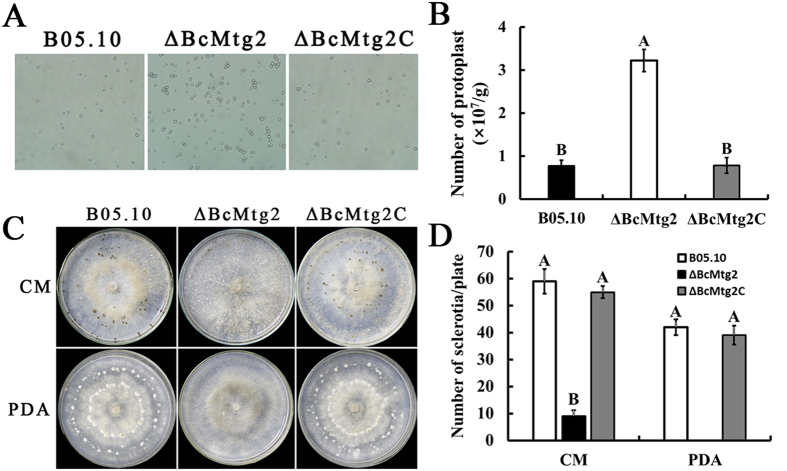
Impact of *BcMtg2* deletion on cell wall lyase sensitivity and sclerotial formation. (**A**) Comparison of protoplast among B05.10, *ΔBcMtg2* and *ΔBcMtg2C* After mycelia incubated at 30 °C for 45 min in 1.5% lyase buffer. (**B**) The number of protoplast release by each strain on lyase buffer. Bars in each column denote the standard errors of three experiments. Values on the bars followed by the same letter are not significantly different at P = 0.05. (**C**) Comparison of sclerotia formation among B05.10, *ΔBcMtg2* and *ΔBcMtg2C* after cultivated on CM and PDA medium for 4 weeks at 25 °C in darkness. (**D**) The number of sclerotia was counted on both CM and PDA. Bars denote standard errors from three experiments. Values on the bars followed by the same letter are not significantly different at P = 0.05.

The *BcMtg2* deletion mutant exhibited increased sensitivity to a cell wall-damaging factor (Congo red). To further understand the role of *BcMtg2* in cell wall integrity (CWI), we analyzed the sensitivity of *ΔBcMtg2* to cell wall lyase. 0.1 g fresh mycelia of each strain was incubated with 15 mL 1.5% lyases buffer (0.6 M KCl, 50 mM CaCl_2_) at 30 °C for 45 min. Then, the amount of protoplast was counted with a hemocytometer. The result showed that *ΔBcMtg2* mycelia were well digested and released significantly more protoplasts than the parental strain B05.10 and the complement strain *ΔBcMtg2*C (Fig. 5A,B), indicating that the *BcMtg2* plays an important role in regulating CWI of *B. cinerea*.

### Effects of *BcMtg2* deletion on sclerotia formation

To investigate the function of *BcMtg2* in sclerotia development in *B. cinerea*, strains were cultivated in the dark for 4 weeks. *ΔBcMtg2* produced significantly fewer sclerotia than B05.10 and *ΔBcMtg2C* on CM medium. In addition, B05.10 and *ΔBcMtg2C* produced vast sclerotia, while the *BcMtg2* deletion mutant did not produce any sclerotia on PDA medium (Fig. 5C,D). These results indicated that *BcMtg2* had an important effect on sclerotial formation in *B. cinerea.*

### *BcMtg2* is required for the virulence of *B. cinerea*

**Figure 6:**
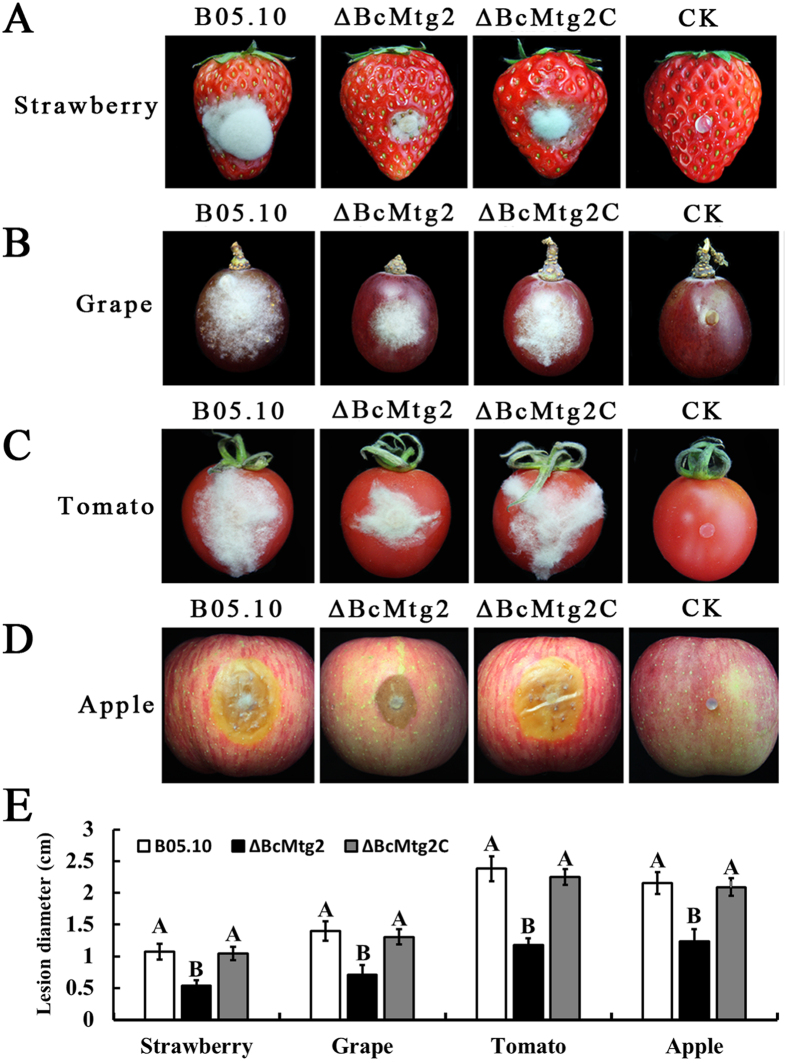
Virulence analysis on plant fruit, following inoculation with the parental strain B05.10, *ΔBcMtg2* and *ΔBcMtg2C*. Agar plugs were used as negative controls (CK). (**A**) Disease symptoms on wounded strawberry fruits after 72 h inoculation (h.p.i.), (**B**) wounded grape fruits 72 h.p.i, (**C**) wounded tomato fruits wounded 72 h.p.i, (**D**) wounded apple fruits 72 h.p.i. (**E**) Diameter of disease lesion on various fruit. Bars denote the standard errors of four repeated experiments. Values on the bars followed by the same letter are not significantly different at P = 0.05.

**Figure 7:**
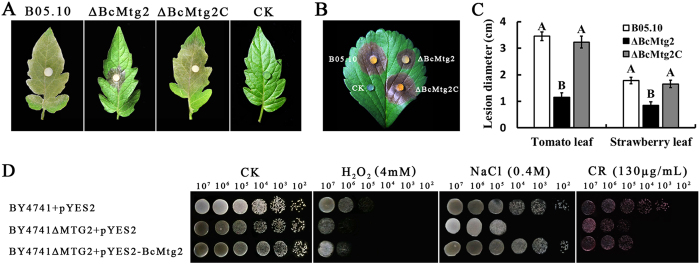
Virulence assays of each strain on plant leaf and complementation of the *S. cerevisiae* MTG2 mutant with *BcMtg2*. (**A**) Disease symptoms on wounded tomato leaves 60 h after inoculation (h.p.i.). (**B**) Disease symptoms on wounded strawberry leaves 60 h after inoculation (h.p.i.). (**C**) Diameter of disease lesion on various plant leaf. Bars denote the standard errors of four repeated experiments. Values on the bars followed by the same letter are not significantly different at P = 0.05. (**D**) Serial dilutions of cell suspensions of each strain were spotted onto YPRG plates including no inhibitor (CK), H_2_O_2_, NaCl and Congo red (CR). The growth of each strain on the plates was examined after yeast cells had been cultivated at 30 °C for 4 days.

To investigate the involvement of *BcMtg2* in virulence, the ability of the strain to infect various plant organisms was evaluated. As indicated in Fig. 6, *ΔBcMtg2* caused significantly smaller disease spots than the parental strain B05.10 and the complement strain *ΔBcMtg2C* on wounded strawberry, grape, tomato and apple fruits, after 72 h of inoculation. Moreover, on wounded tomato leaves and strawberry leaves *ΔBcMtg2* also caused obviously smaller disease spots than B05.10 and *ΔBcMtg2C* after 60 h of inoculation (Fig. 7A–C). These results indicated that *BcMtg2* owned significant effect on the virulence in *B. cinerea*.

### Complementation of yeast *Mtg2* deletion mutants with *BcMtg2*

To further investigate the functions of *BcMtg2*, we determined whether *BcMtg2* could complement the phenotype of the yeast *Mtg2* mutant. Base on the *BcMtg2* deletion mutant shown increased sensitivity to osmotic pressure (NaCl), cell wall inhibitor (Congo red) and oxidative stress (H_2_O_2_), we used the same environment stresses in to analyze the strains growth phenotype in the yeast complement assay. The parental strain and yeast *Mtg2* mutant grew well on YEPG medium. However, compared with the parental strain and the complement strain, the *Mtg2* mutant was obviously inhibited when the culture medium was modified with 4 mM H_2_O_2_ and 0.4 M NaCl. Additionally, the *Mtg2* mutant and the complement strain were significantly hindered when the medium was modified with 130 mg ml^−1^ Congo red. These results indicated that the growth defects of *Mtg2* were partially recovered by genetic complementation of the yeast *Mtg2* mutant with *BcMtg2* (Fig. 7D).

## Discussion

Previous studies showed that the nuclear gene *Mtg2* played an important role in the assembly of mitochondrial ribosomes in *S. cerevisiae*^12^,^18^. The protein is member of the Obg GTPase family^19^. The assembly of cytoplasmic ribosomes requires the coordinated action of assembly proteins^26^. These assembly factors include nucleases, ATP-dependent RNA helicases, modifying enzymes, and a number of recently identified GTPases^26^,^27^,^28^,^29^. To investigate the function of *BcMtg2* in *B. cinerea*, we analyzed the phenotype of the *BcMtg2* deletion mutant. The previous study showed that a deletion mutant of the *Rho4* GTPase gene was defective in vegetative growth and conidiation in *Neurospora crassa*^30^. In our study, the *BcMtg2* deletion mutant exhibited a clear growth defect on MM medium (Fig. 1A). Moreover, compared with the parental strain and the complement strain, *ΔBcMtg2* produced significantly fewer conidia and exhibited a lower conidia germination rate. The previous study indicated that the Ras small GTPase was involved in sclerotia development in *Sclerotinia sclerotiorum*^31^. The previous study showed that sclerotial development plays a significant role in the survival mechanism of *B. cinerea* in dying host organisms^21^. In our study, we found that *ΔBcMtg2* produced significantly fewer sclerotia than the parental and complemented strains. In addition, in our study, we found that the *BcMtg2* deletion mutant exhibited decreased virulence in various plant tissue. A similar phenotype was also observed in a *Rho3* GTPase deletion mutant in *B. cinerea*^32^. These consequences indicated that GTPases may have a similar function in the regulation of asexual reproduction, vegetative growth, survival mechanism and virulence in plant pathogenic fungi.

In our study, the *BcMtg2* deletion mutant exhibited significantly decreased virulence on host plant tissue. To date, the exact causes of the decreased pathogenicity of the *BcMtg2* deletion mutant remain unclear, we inferred that the decreased pathogenicity of *ΔBcMtg2* might be closely related to its reduced tolerance to various environmental stresses. First, the deletion of *BcMtg2* leads to increased sensitivity of *B. cinerea* to cell wall-disrupting factors. The previous studies had shown that cell wall integrity is required for *B. cinerea* virulence^33^,^34^. Second, the *BcMtg2* deletion mutant appeared to have increased sensitivity to oxidative stresses, which can be generated by a host plant during the host-fungus interaction^35^,^36^. However, *B. cinerea* possesses an effective ROS detoxification mechanism to address an oxidative burst^37^. The *BcMtg2* deletion mutant may defect in ROS eliminated ability which resulted in the decreased pathogenicity. Third, a previous study showed that the fungal infection process might be related to osmotic stress^38^,^39^. The increased sensitivity of *ΔBcMtg2* to osmotic stress may lead to defective virulence of *B. cinerea* on host plant tissue. Osmotic, oxidative stresses generated by host plant and cell wall integrity of *B. cinerea* significantly effect on the interaction of *B. cinerea* with host plant tissue^33^,^34^,^35^,^36^. To analyze the sensitivity of *B. cinerea* to osmotic, oxidative stresses, and to Congo red which can enhance our understanding the reasons for the defect of *BcMtg2* deletion mutant on virulence.

In our study, *ΔBcMtg2* exhibited increased sensitivity to various environmental stresses compared with the parental and complemented strains. In the cell wall tolerance and osmotic pressure assays, we found that *ΔBcMtg2* showed increased sensitivity to Congo red but not caffeine and increased sensitivity to Na but not K, respectively. This result may be caused by the fact that *ΔBcMtg2* is selective between Congo red and caffeine and has the ability to adapt to different osmotic pressures generated by Na or K. This hypothesis needs to be explored in the future. Additionally, in yeast complement assay, we found the *BcMtg2* was able to partially complement the phenotype defects of yeast *Mtg2* mutant under the various environment stresses (NaCl, Congo red and H_2_O_2_). We deduced that the sequence difference between *BcMtg2* and *ScMtg2* leads to the biological function variation (Fig. S1). This assumption needs to be explore in the future research.

In conclusion, these results have indicated that the *BcMtg2* protein possesses an important role in pathogenicity, vegetative growth, asexual reproduction, and tolerance to multiple environmental stresses. Additionally, the *BcMtg2* deletion mutant may influence the function of mitochondrial ribosomes to cause the different phenotypes we observed in our study. This assumption is being investigated in our lab. Based on the conclusions in our study, the *Mtg2* protein played an important role in growth and development in *B. cinerea*. This protein was hopeful as a new potential antifungal drug target for the development of novel fungicide against gray mold caused by *B. cinerea*.

## Methods

### Strains, media and growth conditions

*Botrytis cinerea* strain B05.10 was isolated from a grape in California, USA used as the wild-type progenitor for the construction of the gene deletion mutant in this study^32^. The wild-type strain B05.10 and mutant strains were grown on PDA, MM or CM^37^ for the mycelial growth assays. For stress tests, strains were cultured on PDA modified with various supplements. Each plate was inoculated with a 5-mm diameter mycelial plug taken from the margin of a 3-day-old colony grown on PDA. In the yeast complement assay, all strains were grown on YEPG (1% yeast extract, 2% peptone, 1% galactose, 1% raffinose and 2% agar)^37^. The experiments were repeated three times independently.

### Identification of *BcMtg2* in *B. cinerea*

Based on the *B. cinerea* genome sequence database (http://www.broadinstitute.org/), *BcMtg2* (BC1G_14222) was initially identified using the BLASTP algorithm and the *Mtg2* protein from *S. cerevisiae* as the query. To determine the size and existence of introns, RNA was extracted from the hypha of the parental strain B05.10 using the RNAsimple Total RNA Kit (Tiangen Biotech. Co., Beijing, China) and used for first-strand cDNA synthesis with the PrimeScript RT Reagent Kit (TaKaRa, Dalian, China). PCR amplification of the cDNA was performed with the P1/P2 primer pair (Table S1). The resultant PCR product was purified, cloned and sequenced.

### Generation of the *BcMtg2* deletion mutant

The *BcMtg2* deletion vector was constructed as described previously^40^. First, fragments 1.4 kb upstream and 1.3 kb downstream of *BcMtg2* were amplified from the genomic DNA of the parental strain B05.10 using the primer pairs P3/P4 and P5/P6 (Table S1, Fig. S2A), respectively. The primer pair P7/P8 was used to amplify a 1.7-kb *HPH* cassette containing the hygromycin resistance gene and the *Aspergillus nidulans trpC* promoter^41^. Then, the three amplicons (upstream, *HPH* cassette and downstream) were fused using the primer pair P9/P10^42^ (Table S1, Fig. S2A). The resultant PCR product was gel purified with the AxyPreTP DNA Gel Extraction Kit (Axygen, USA) and used to transform protoplasts of the parental strain B05.10. Protoplasts were prepared, and transformation was performed as described previously^43^.

### Complementation of the *BcMtg2* deletion mutant

To confirm that the defects of the *BcMtg2* deletion mutant were caused by the gene deletion, the *BcMtg2* deletion mutant was complemented with the entire *BcMtg2* gene. The complemented *BcMtg2* gene was amplified from the genome of the parental strain B05.10 using the P20/P21 primer pair, which included the 325-bp promoter sequence and 543-bp terminator region (Table S1). The resultant PCR product was cloned into the *SmaI-XbaI* site of pNEO to generate the complement vector, pNEO-BcMtg2-C. *BcMtg2* in this vector was sequenced to verify that no errors were present in this sequence before the complement was transformed into the *BcMtg2* deletion mutant. The transformation of *ΔBcMtg2* with the complement vector was performed as previously described^40^.

### Southern blot assay

Genomic DNA was extracted from *B. cinerea* using the previously described method^44^ and digested with *XhoI*. Southern blot analysis was performed with the 241-bp downstream fragment of *BcMtg2* as a probe. The fragment was amplified from the B05.10 genome using the primer pair P23/P24 (Table S1, Fig. S2A). The probe was labeled with digoxigenin (DIG) using the High Prime DNA Labeling and Detection Starter Kit II (Roche Diagnostics).

### Conidiation and sclerotial development analysis

To evaluate conidiation, mycelial plugs (5 mm in diameter) obtained from the margin of a 3-day-old colony were cultivated on PDA medium and grown at 25 °C for 8 days. Spores from each plate were acquired using sterile H_2_O. The suspension was filtered using three layers of gauze, and the number of spores was calculated by a hemocytometer. To assay sclerotial production, 5-mm diameter mycelial plugs taken from the edge of a 3-day-old colony of each strain were inoculated onto PDA or CM and cultivated at 25 °C for 4 weeks in the dark. Then, the number of sclerotia was measured using a previously described method^45^. The experiment was repeated three times.

### Assays for glycerol accumulation

To determine intracellular glycerol content, each strain was cultivated in YEPD medium (w/v, 1% peptone, 0.3% yeast extract, 2% glucose) for 2 days at 175 rpm at 25 °C in a shaker. After supplementing with 1.0 M NaCl and further incubation for 2 h, the mycelia of each strain were collected and ground in liquid nitrogen. Then, the mycelial powder (150 mg) was transferred to a 2-ml microcentrifuge tube containing 1 ml of glycerol extraction buffer (Applygen Technologies Inc., Beijing, China). After vortexing sufficiently for 1 min, the tubes were centrifuged at 6000× g for 10 min. Then, 10 μL of the resulting supernatant of each sample was transferred to a new tube, and a 5-μL aliquot of each supernatant was mixed with 190 μL of glycerol determination buffer (Applygen Technologies Inc., Beijing, China). The coloration process was cultivated at 37 °C for 15 min. Then, the glycerol content was measured with a microplate reader (Molecular Devices Inc.) at 550 nm^37^. The experiment was independently repeated three times.

### Quantitative real-time PCR

To extract total RNA, the mycelial plugs of the wild-type strain B05.10, the *BcMtg2* deletion mutant or the complemented strain *BcMtg2C* were transferred to YEPD medium and cultured for 2 days at 25 °C in dark. Before the mycelia were harvested for RNA extraction, the culture was treated for 2 h with NaCl or Congo red at the concentrations indicated in the legend of Fig. 4. Total RNA was extracted from the mycelia of each strain using the RNeasy Kit (Tiangen Biotech. Co). Then, total RNA of each strain was purified using DNase to remove the contaminated DNA with Reagent Kit (TaKaRa), and 10 μg of each RNA sample was used for First Strand cDNA synthesis with the PrimeScript® RT Reagent Kit (TaKaRa). Real-time PCR was conducted in an ABI 7500 real-time system (Applied Biosystems) using SYBR Green I fluorescent dye detection. Amplification was conducted in a 20 μL volume containing 10 μL of iTaq™ Universal SYBR® Green Supermix (Bio-Rad Laboratories), 2 μL of the reverse transcription product and 1 μL each of the forward and reverse primers (Table S1). There were three replicates for each sample. The expression of the measured genes in each sample was normalized to the expression level of reference gene *actin*, and relative changes in gene expression levels were analysed with the ABI 7500 SDS software (Applied Biosystems), which automatically sets the baseline. Data from three biological replicates were used to calculate the mean and standard deviation^46^. The experiment was repeated three times.

### Determination of the sensitivity of *ΔBcMtg2* to environmental stress

Mycelial growth assays were performed on PDA plates modified with the following elements: Congo red, caffeine, KCl, NaCl, H_2_O_2,_ and paraquat at the concentrations shown in the figures. The percentage of inhibition of mycelial radial growth (PIMG) was calculated using the formula, PIMG = [(C − N)/(C − 5)] × 100, where C is the colony diameter of the untreated control and N is that of stress treatment^47^. The experiments were repeated twice.

### Yeast complementation assays

The yeast wild type strain BY4741 and the MTG2 deletion mutant BY4741ΔMTG2 was provided by EUROSCARF (http://web.uni-frankfurt.de/fb15/mikro/euroscarf/). The full-length *BcMtg2* cDNA was amplified from total RNA of B05.10 using the primer pair P35/P36 (Table S1). The PCR product was digested with *BamHI* and *XbaI* and cloned into the pYES2 vector (Invitrogen), and transformed into the yeast mutant BY4741ΔMTG2. Yeast transformants were selected on synthetic medium lacking uracil (Clontech). Additionally, the wild-type strain BY4741 and BY4741ΔMTG2 mutant transformed with empty pYES2 vector were used as controls. In the complementation assays, the yeast transformants were grown at 30 °C for 4 days on YPRG medium (1% yeast extract, 2% peptone, 1% galactose, 1% raffinose, 2% agar) modified with various stress agents including Congo red, NaCl, and H_2_O_2_ at concentrations shown in figure legends. The experiments were repeated three times independently.

### Plant tissue infection assays

Five-millimeter diameter mycelial plugs of 3-day-old colony grown on PDA were inoculated onto fully expanded leaves of tomato and strawberry plants, and grape, tomato, strawberry and apple fruits. Inoculated plant tissues were cultivated at 25 °C with 16 h of daylight^45^. The disease lesions were recorded after the indicated times in the figure legends. The experiment was repeated four times.

## Additional Information

**How to cite this article**: Shao, W. *et al*. *BcMtg2* is required for multiple stress tolerance, vegetative development and virulence in *Botrytis cinerea*. *Sci. Rep.* **6**, 28673; doi: 10.1038/srep28673 (2016).

## Supplementary Material

Supplementary Information
