# Supplementary material for: BcMtg2 is required for multiple stress tolerance, vegetative development and virulence in Botrytis cinerea
Source: Sci Rep. 2016 Jun 27;6:28673. doi: 10.1038/srep28673 (PMC4921815; doi:10.1038/srep28673)
Supplement: Supplementary Information [file srep28673-s1.pdf]

Scientific Report

BcMtg2 is required for multiple stress tolerance, vegetative development and virulence in *Botrytis cinerea*

Wenyong Shao, Yu Zhang, Jin Wang, Chiyuan Lv and Changjun Chen*

College of Plant Protection, Nanjing Agricultural University,
Nanjing 210095, Jiangsu, China
Corresponding author: Changjun Chen
Phone: 086-25-84395641;
Fax: 086-25-84395641;
E-mail: changjun-chen @njau.edu.cn

Figure S1

Amino acid alignments of Botrytis cinerea Mtg2 (BcMtg2) with those of Neurospora crassa Mtg2 (NcMtg2), Candida albicans Mtg2 (CaMtg2), Aspergillus nidulans Mtg2 (AnMtg2), Magnaporthe oryzae Mtg2 (MoStr), Fusarium graminearum Mtg2 (FgMtg2), Fusarium oxysporum (FoMtg2), Schizosaccharomyces pombe Mtg2 (SpMtg2), Saccharomyces cerevisiae Mtg2 (ScMtg2) and Sclerotinia sclerotiorum Mtg2 (SsMtg2). Boxshade program was used to highlight identical (black shading) and similar (grey shading) amino acids. The conserved N-terminal pyridoxal phosphate binding pocket and the C-terminal catalytic residue domain are underlined. Phylogenetic relationship of the Mtg2 gene among Botrytis cinerea and other plant pathogens based on amino acid sequence.

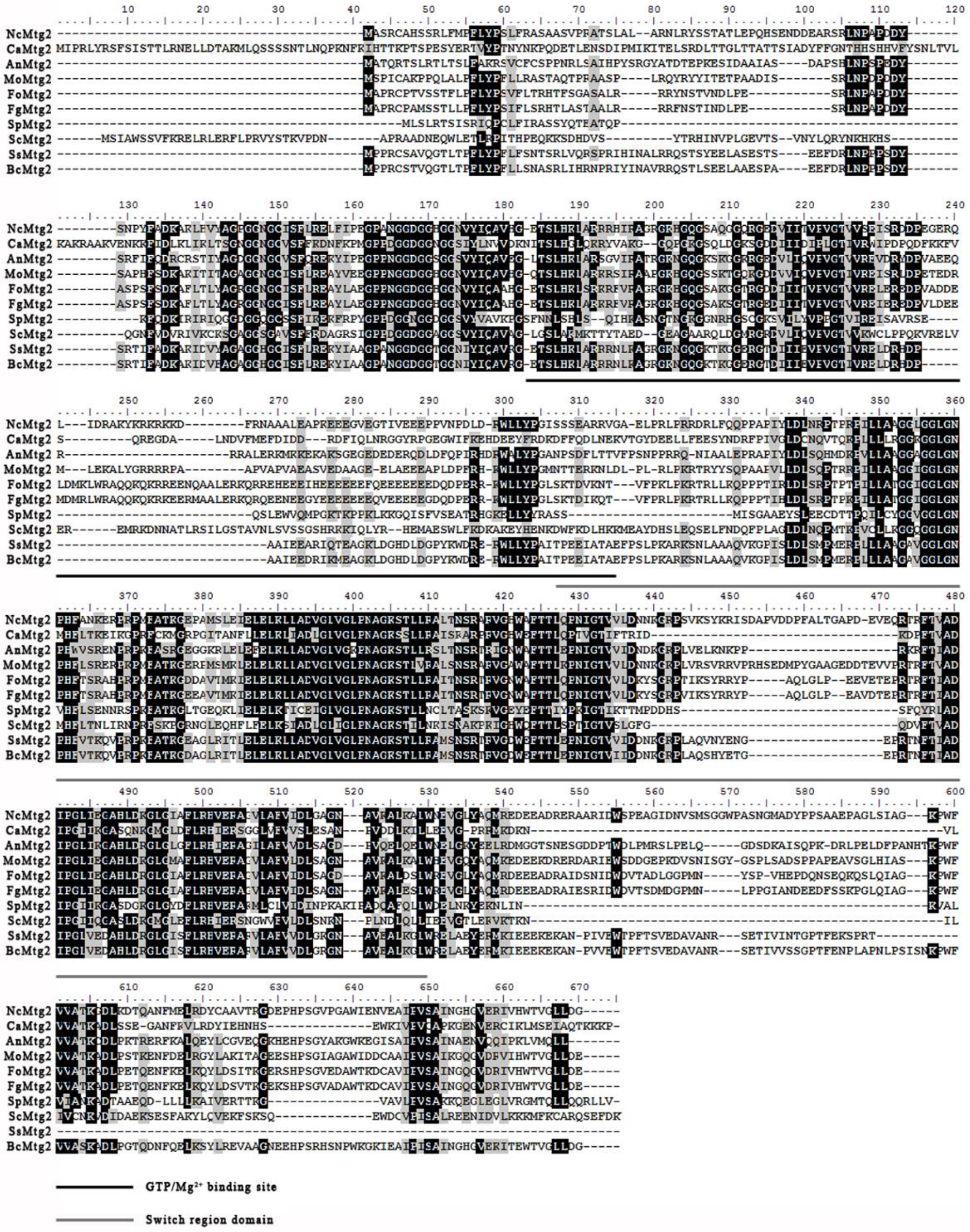
GTP/Mg2+ binding site
Switch region domain

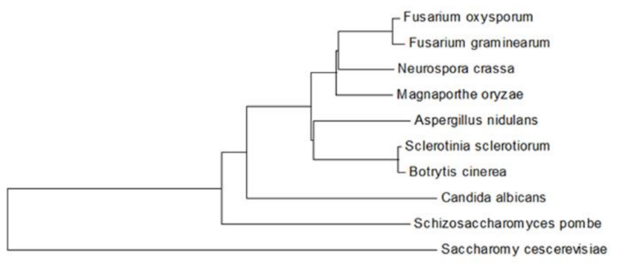
Fusarium oxysporum
Fusarium graminearum
Neurospora crassa
Magnaporthe oryzae
Aspergillus nidulans
Sclerotinia sclerotiorum
Botrytis cinerea
Candida albicans
Schizosaccharomyces pombe
Saccharomy cescerevisiae

Figure S2

Generation and identification of the BcMtg2 deletion mutant of Botrytis cinerea. (A) Gene replacement strategy for BcMtg2. The hygromycin resistance cassette (hph) is denoted by the large grey arrow. Primer (P3–P18) binding sites are indicated by arrows (see Table S1 for the primer sequences). (B) Southern blot hybridization analysis of strains using the 3′-flanking region of BcMtg2 as a probe. Genomic DNA of the wild-type progenitor B05.10, BcMtg2 deletion mutant ΔBcMtg2 and complemented strain ΔBcMtg2C were digested with XhoI. (C) Primer pair P11/P12 was used to specifically amplify the partial BcMtg2. (D) Primer pair P13/P14 was used to validate the selectable marker hph. (E, F) Primer pairs P15/P16 and P17/P18 were used to amplify the two homologous arms with a partial fragment of the connecting area.

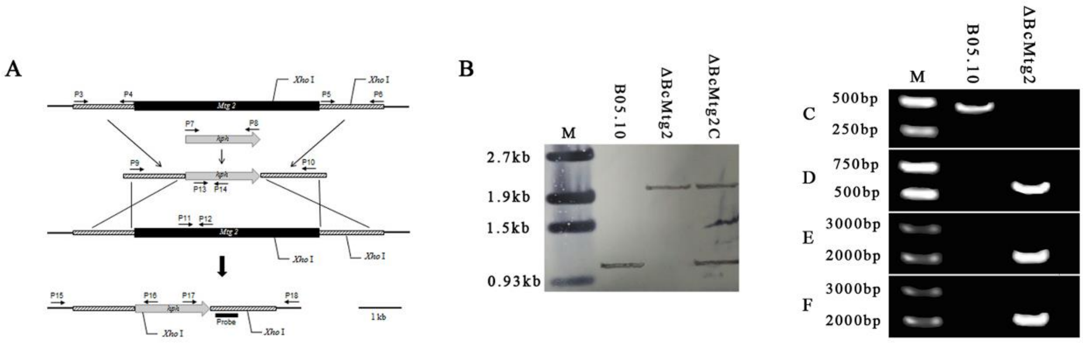

Table S1
Primers used in this study

Primer code Sequence (5′→3′)	Relevant characteristic
P1 ATGCCTCCACGTTGTTCAAC	Amplify the full cDNA sequence of the *Mtg2* gene
P2 TCATCCATCAAGAAGACCAAC	
P3 TTCGTATGAAGCCGTATGT	Amplify the left homologous arm of the *Mtg2* gene of *B. cinerea* (1444 bp)
P4CCACCAGCCAGCCAACAGCTCCCAGCTGTGCGAGCTGGAGA	
P5 CAATACGCAAACCGCCTCTCCCCAGTATTTACGGATAGAGGC	Amplify the right homologous arm of the *Mtg2* gene of *B. cinerea* (1517 bp)
P6 CAATGAGTTATGGAGGTG	
P7 GGGAGCTGTTGGCTGGCTGGTGG	Amplify the *hph* gene (1764 bp)
P8 GGGGAGAGGCGGTTTGCGTATTG	
P9 AATGGTGAAGGCTCTGCTGT	Amplify the knockout vector of the *Mtg2* gene of *B. cinerea* (4471 bp)
P10 ATGAGTTATGGAGGTGGGT	
P 11 AGTTTACCACATTGGAGCCG	Amplify a partial fragment of the *Mtg2* gene of *B. cinerea* (437 bp)
P 12 GGGAGATTAGGTGCGAGGG	
P 13 CAAAGCATCAGCTCATCGAGAG	Amplify a partial fragment of the *hph* gene (503 bp)
P 14 GAAAAGTTCGACAGCGTCTCC	
P 15 GGGTAGTTGGCATGAAAG	Confirm whether the *hph* genes homologously replaced the *Mtg2* gene of *B. cinerea* (2154bp)
P 16 GTACTCGCCGATAGTGGA	
P 17 TCATTTGGATGCTTGGGTAG	Confirm whether the *hph* genes homologously replaced the *Mtg2* gene of *B. cinerea* (2049bp)

P 18 CTTATGGATCTCGGTAGTGC	
P19 CTGCAGAAccaccatgttggGTCGACAGAAGATGATATTG	Amplify the NEO cassette containing a trpC promoter (1181 bp)
P20 CCGctcgagTCAGAAGAACTCGTCAAGAAGGCG	
P21 TCCcccgggGGAATTTGGTTTCCGAGGTA	Amplify the *Mtg2* gene (include the control region of the *Mtg2* gene) (2614 bp)
P22 GCtctagaGC ATCCAGCGTTTCGTGTA	
P23 TATCTGTCACAAGTGGCGTAT	Amplify a probe for Southern blotting (241 bp)
P24 AAGAATGTTCCTTCCTCCC	
P25 TAGGTGATTTGGGACAACAGAG	Amplify the *BcBos1* gene for quantitative real-time PCR
P26 GTCTCTCAATGGTGCGGATAG	
P27 GAAGGATGGAAGGGTCTTGTAG	Amplify the *BcSAk* gene for quantitative real-time PCR
P28 CTAACTCGGAGAAAGCCTGATG	
P29 GCTACGCCTATGGGAAGTAATG	Amplify the *BcMkk1* gene for quantitative real-time PCR
P30 CTACTTTCGCTTCCTCCACTTG	
P31 GGCAAGTTGAAGGAGCAAATC	Amplify the *BcGls* gene for quantitative real-time PCR
P32 ATCTGGTGCGTGTGTGATAG	
P33 CGTCTGGATTGGTGGTTCTATT	Amplify the reference gene actin for quantitative real-time PCR
P34 ACTCGTCGTACTCTTGCTTTG	
P35 CGggatccCGATGCCTCCACGTTGTTCAAC	the full cDNA sequence of the *BcMtg2* gene for construction of pYES2-BcMtg2
P36 GCtctagaGCTCATCCATCAAGAAGACCAAC	
